# Spontaneous emission rate enhancement with aperiodic Thue-Morse multilayer

**DOI:** 10.1038/s41598-019-44901-0

**Published:** 2019-06-11

**Authors:** Ling Li, Cherian J. Mathai, Shubhra Gangopadhyay, Xiaodong Yang, Jie Gao

**Affiliations:** 1 Department of Mechanical and Aerospace Engineering, Missouri University of Science and Technology, Rolla, Missouri 65409 United States; 2 Department of Electrical and Computer Engineering, University of Missouri, Columbia, Missouri 65211 USA

**Keywords:** Metamaterials, Quantum dots

## Abstract

The emergence of multilayer metamaterials in the research field of enhancing spontaneous emission rates has recently received extensive attention. Previous research efforts mostly focus on periodic metal-dielectric multilayers in hyperbolic dispersion region; however, the influence of lattice order in subwavelength multilayers on spontaneous emission is rarely studied. Here, we observe the stronger Purcell enhancement of quantum dots coupled to the aperiodic metal-dielectric multilayer with Thue-Morse lattice order from elliptical to hyperbolic dispersion regions, compared to the periodic multilayer with the same metal filling ratio. This work demonstrates the potential of utilizing quasiperiodic metamaterial nanostructures to engineer the local density of states for various nanophotonic applications.

## Introduction

Since the discovery of Purcell effect of a resonant cavity on spontaneous emission rate of single atoms in 1946^1,2^, modification of spontaneous emission rate of quantum emitters has become a fundamental research direction in the field of quantum electrodynamics^3–5^, and also very important for technological advances, such as single-photon sources^6–9^, light-emitting devices^10–13^, low-threshold photonics^14–16^, and plasmon lasers^17–20^. Following the advances in micro- and nano-fabrication technologies, there has been a surge of works on exploiting the electromagnetic resonance of microcavities^21–24^, photonic crystals^25–28^, nanoparticles and nano-antennas to enhance spontaneous emission^29–32^. Optical metamaterials, a unique kind of artificial structure, emerged and received an enormous amount of attention due to their capabilities to realize extraordinary electromagnetic properties rare or absent in naturally occurring materials. Recently, extensive research efforts on one class of metamaterials called hyperbolic metamaterials demonstrated anomalous electromagnetic phenomena such as negative refraction, subwavelength imaging, the diverging local density of states, epsilon-near-zero (ENZ) and optical nonlocality^33–44^. Unlike the resonant structures, hyperbolic metamaterials are capable of providing broadband Purcell effect^44–46^.

Hyperbolic metamaterials based on multilayer structures, consisting of an alternating arrangement of metal and dielectric layers, provide significant platforms for enhancing spontaneous emission and other unique electromagnetic properties. A series of experiments demonstrated the capability of metamaterials in enhancing spontaneous emission of various quantum emitters such as dye molecules, nitrogen-vacancy centers, semiconductor nanocrystals, and colloidal quantum dots (QDs)^13,30,44,45,47–51^. In the hyperbolic region, the strongly coupled surface plasmon polaritons (SPPs) on metal-dielectric interfaces of the multilayer metamaterials, enabling optical modes with anomalously large wave vectors, tremendously modify the local density of states (LDOS). The majority of multilayers explored in this regard so far are periodic multilayers (PM). For aperiodic multilayers, Moritake *et al*. showed a 1.35 fold Purcell factor enhancement of a multilayer based on Fibonacci sequence than that of the PM at one wavelength with hyperbolic dispersion^52^. In this work, we have investigated a PM (4 periods, 8 layers) and a deterministic aperiodic multilayer of Thue-Morse (ThM) sequence (3^th^ generation, 8 layers) to study the effect of lattice order on the spontaneous emission rate of CdSe/ZnS QDs on top of the multilayers. Compared to PM with the same filling ratio, a stronger reduction of photoluminescence decay time of QDs on ThM was experimentally observed across a broad wavelength band of 70 nm. Theoretical analysis shows that QDs on ThM experienced stronger LDOS enhancement than that on PM from elliptical to hyperbolic dispersion regions. A clear distinction between dipole-excited electric field distributions inside the two types of multilayer stacks is also found in numerical simulation. The field distribution spreads out inside ThM indicating a stronger interaction between the dipole and coupled SPP modes. This work demonstrates the LDOS engineering capability of an aperiodic multilayer metamaterial with both experimental characterization and theoretical analysis, which can further extend the potential of tailoring light-matter interactions with artificial nanostructures.

## Results and Discussion

**Figure 1:**
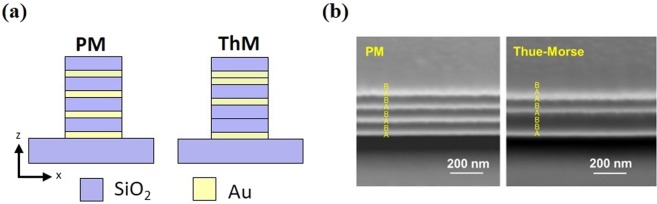
(**a**) Schematic of the Au-SiO_2_ periodic multilayer (PM) and Thue-Morse multilayer (ThM) stack. (**b**) The SEM pictures of the cross-section of the fabricated 4-pair Au-SiO_2_ PM and third generation ThM multilayer stacks.

We fabricated the one-dimensional ThM multilayer stack by stacking two different materials A and B, following a deterministic inflation scheme: A → AB, B → BA, with A as an initiator stacking from the silica substrate. Figure 1(a) depicts the schematic of a 4-pair PM metal-dielectric multilayer stack and the third generation sequence of ThM aperiodic multilayer stack, ABBABAAB, where A and B represent gold (Au) and silica (SiO_2_) layer with a designed thickness of 20 and 80 nm, respectively. The ThM multilayer is designed to have the same metal filling ratio of 0.2 as the PM multilayer. For QDs photoluminescence measurements, we diluted the original CdSe/ZnS QDs solution with the mixed solution of polymethyl methacrylate, anisole, and chloroform. The diluted solution of QD-PMMA has a center photoluminescence wavelength around 604 nm and was then spin-coated on surfaces of multilayers and a glass substrate serving as a control. The thickness of a coated PMMA matrix layer was estimated to be around 50 nm from our past experience^53^.

**Figure 2:**
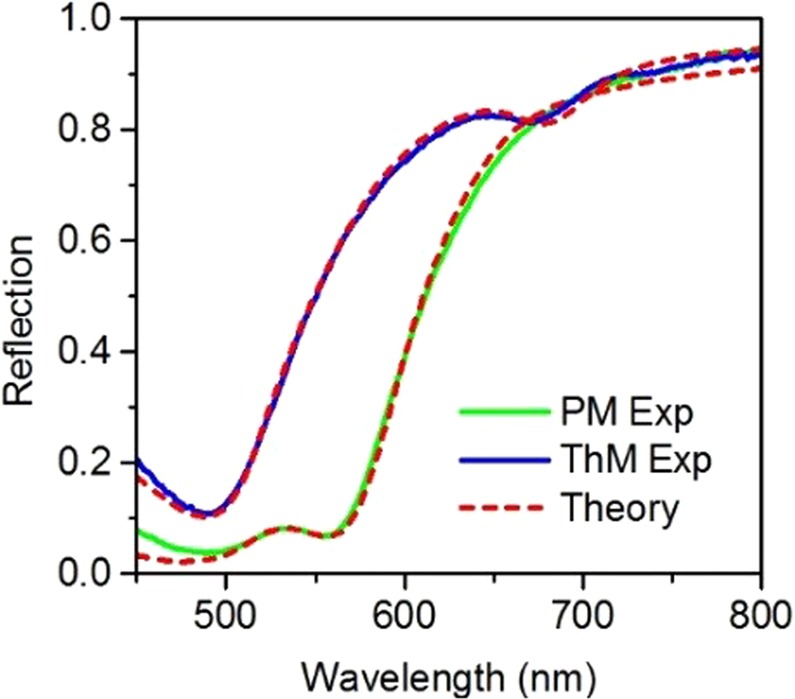
Measured reflection spectra of the Au-SiO_2_ PM and ThM multilayer stacks.

Normal-incidence reflection spectra of both PM and ThM multilayer stacks were measured in the wavelength range of 450 nm to 800 nm. The measured spectra are shown in Fig. 2 along with the theoretical fitting using the transfer matrix method. The extracted Au and SiO_2_ layer thickness are 20 nm (20 nm) and 79 nm (80 nm) for PM (ThM) multilayer, respectively. These fitted layer thicknesses as well as the permittivities for Au and SiO_2_ from references^54,55^ used in the reflection spectra fitting were then used in all theoretical analysis and calculations, and the host material is set to be vacuum.

**Figure 3:**
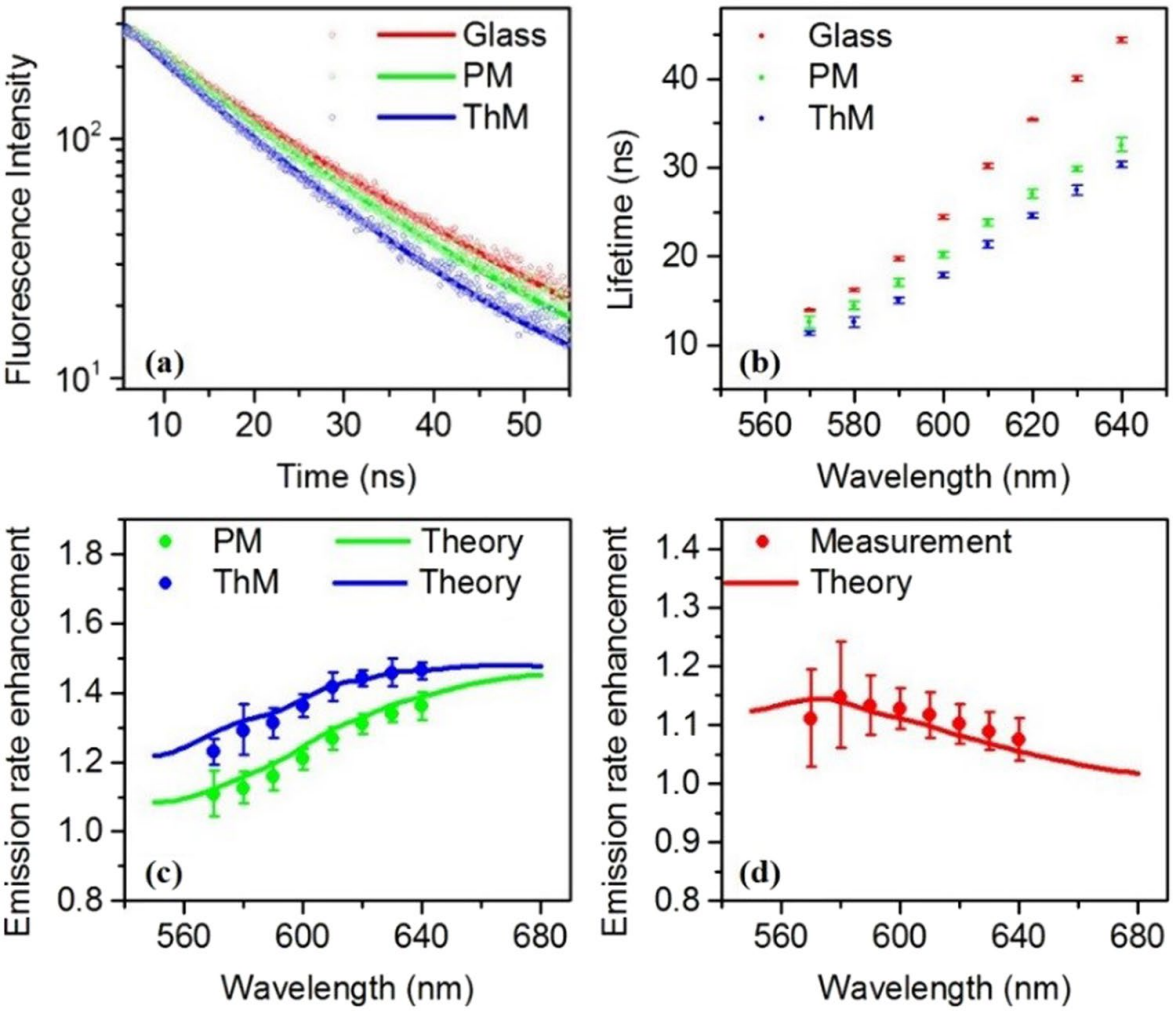
(**a**,**b**) Photoluminescence decay measurements (**a**) and the fitted lifetime data (**b**) for QDs spin-coated on a glass coverslip (red), PM (green), ThM (blue). (**c**) Emission rate enhancement for PM (green solid dots) and ThM (blue solid dots) multilayers deduced from normalizing the lifetime data measured on glass coverslips by those from multilayers. (**d**) Relative emission rate enhancement deduced from normalizing the lifetime data measured on PM by ThM. Experimental data are supported by theoretical calculations denoted by solid lines.

We conducted time-resolved photoluminescence measurements by using a time-correlated single photon counting system. Figure 3(a) shows the photoluminescence decay measurements from the QDs on the two multilayer stacks and the glass substrate at the emission wavelength of 580 nm. The QDs decay lifetimes are obtained from the modified exponential model fitting (shown as the solid lines)^44^ of the photoluminescence decay curves, and the values are 16.2 ns (glass), 14.4 ns (PM) and 12.5 ns (ThM). This modified exponential decay model, $I(t)={I}_{0}{e}^{-{(t/\tau )}^{\beta }}+{I}_{b}$, is basically a stretched exponential relaxation model with an amplitude *I*_0_, background *I*_*b*_, and a stretching parameter *β*. To obtain the photoluminescence lifetime τ, the three parameters were used to fit the measured photoluminescence decay data. The photoluminescence decay lifetimes deduced from the QDs decay measurements within the emission wavelength range of 570 nm to 640 nm are illustrated in Fig. 3(b). The error bar at each wavelength reflects the variation of the measured lifetimes at more than ten different spots on sample surfaces. The lifetime reductions of photoluminescence decay for QDs on both PM and ThM multilayer stacks are stronger than that on the glass substrate, with the most substantial reduction occurred at the longest wavelength of 640 nm. Figure 3(c) presents the measured emission rate enhancement (obtained by normalizing QDs decay lifetime on the glass substrate with that on the multilayers) for both PM and ThM multilayer stacks, which agrees very well with the theoretical Purcell factor calculations shown in solid lines (calculation method in section 4). The calculations take into account realistic QDs quantum efficiency, random dipole polarizations and various dipole positions along *z* direction (averaged from ten positions within 5–50 nm above the surface)^44,56^. The ThM multilayer stack has stronger Purcell effect than PM multilayer stack through the broad range of emission wavelength. Figure 3(d) shows the relative emission rate enhancement from ThM multilayer stack with respect to that of PM stack. The relative emission rate enhancement presents a peak around the wavelength of 580 nm, which also matches the calculation very well. Around this emission wavelength, a larger emission rate enhancement by ThM multilayer stack originates from a stronger dipole-SPPs interaction.

To understand the impact of lattice order on Purcell effect in PM and ThM multilayer stacks, the Purcell factor experienced by a perpendicularly polarized dipole near the multilayer stack surface is theoretically investigated based on the layer thicknesses and permittivities parameters. The corresponding local density of states (LDOS) is in the form of $(3{k}_{0}/2{k}_{z}){(u/\sqrt{{\varepsilon }_{1}})}^{3}(1+{r}_{p}{e}^{2i{k}_{{\rm{z}}}d})$, where *k*_*0*_ is the magnitude of the wave vector in vacuum, *u* = *k*_*x*_/*k*_*0*_ is the normalized wavevector component parallel to multilayer surface, ${k}_{z}={k}_{0}\sqrt{{\varepsilon }_{1}-{u}^{2}}$ is the component of the wavevector perpendicular to the multilayer interface, *ε*_1_ is the relative permittivity for the host material, and *r*_p_ is the reflection coefficient at the interface for a *p*-polarized wave^44,51^.

**Figure 4:**
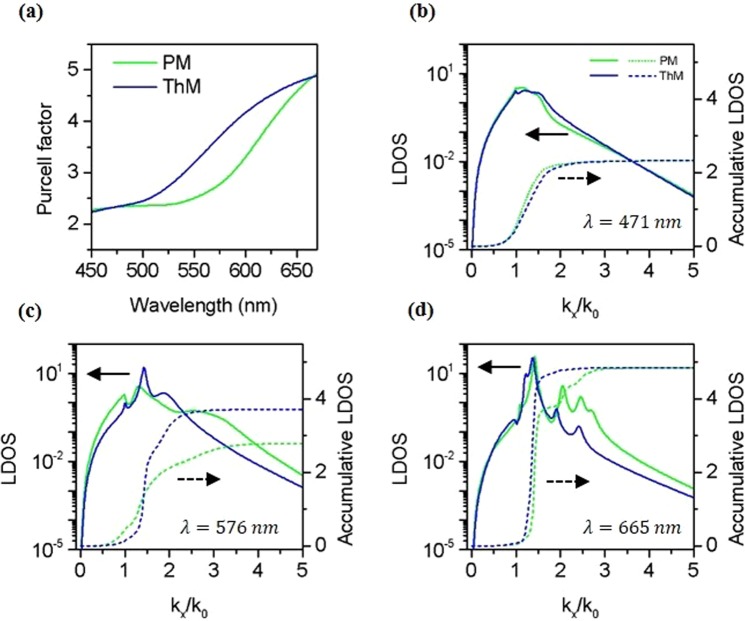
Theoretical Purcell factor, the LDOS and cumulative LDOS for a perpendicularly polarized dipole at a distance of 5 nm above two multilayers in vacuum. (**a**) Purcell factor for the dipole on PM (green) and ThM (blue) multilayer stacks. (**b**–**d**) The LDOS (solid lines) and cumulative LDOS (dashed lines) for the dipole at the emission wavelength of 471 nm (**b**), 576 nm (**c**), and 665 nm (**d**).

Figure 4(a) shows the theoretically calculated Purcell factor of a dipole at a distance of 5 nm above the top surface of multilayer stacks with dipole polarization perpendicular to the multilayers. ThM multilayer stack shows a stronger Purcell effect than PM multilayer stack does in a broad wavelength range from 450 nm to 670 nm. We also observe through theoretical calculations that Purcell factors of both PM and ThM decay exponentially with the dipole-multilayer distance, and reduce to unity when dipole is pulled more than 100 nm above multilayers. From the effective medium theory analysis, the ENZ wavelength for the two multilayers is around 587 nm. The effective permittivity sign change around this wavelength leads to a dispersion transition from closed elliptical to open hyperbolic. The Purcell factor and LDOS in this elliptical-to-hyperbolic transition region are studied to reveal the influence of PM and ThM lattice order on spontaneous emission process. As shown in Fig. 4(b–d), The LDOS and the accumulated LDOS of the dipole are examined at the emission wavelength of 471 nm (elliptical region), 576 nm (near ENZ), and 665 nm (hyperbolic region), as a function of the normalized wavevector component parallel to the multilayer stack surface *k*_*x*_/*k*_0_. The accumulated LDOS is the integration of LDOS up to a specific in-plane wave-vector value, and the value corresponds to the Purcell factor shown in Fig. 4(a) when the upper integration limit is infinity. As shown in Fig. 4(c), the LDOS and accumulated LDOS of ThM multilayer stack at the emission wavelength of 576 nm are clearly larger than those of the PM stack are, resulting in a 1.33 fold enhancement for the Purcell factor. The pronounced LDOS peaks of ThM multilayer at 576 nm (shown in log scale) are observed around the wavevector component *k*_*x*_*~1.5k*_*0*_ compared with PM multilayer, which lead to the enhanced accumulative LDOS and Purcell factor for ThM multilayer. According to Fig. 4(c), the far-field radiation for PM is stronger than ThM in the *k*_*x*_*/k*_*0*_ < 1 region, but the total decay rate enhancement for the aperiodic ThM is larger than PM. This indicates that the dipole emitters coupled to ThM multilayer experience a strong non-radiative decay enhancement. Indeed, the measured fluorescence intensity of quantum dots on PM is brighter than that of ThM.

**Figure 5:**
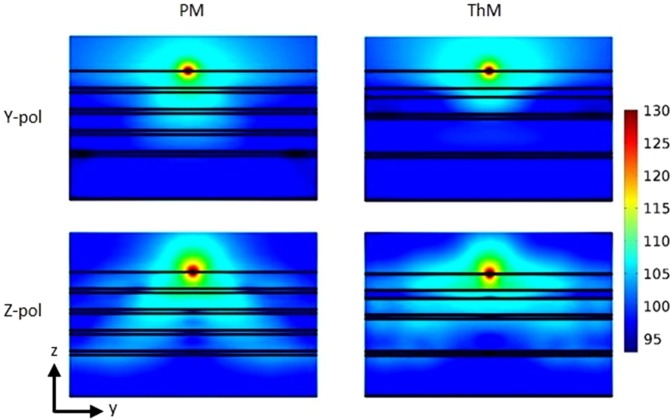
Electric field intensity distributions (on binary logarithmic scale) inside both multilayer stacks excited from a dipole emitter 5 nm above the multilayer top surface with an emission wavelength of 576 nm. The top and bottom row show the intensity distribution for the *y*-polarized and *z*-polarized dipolar emitter, respectively.

A close comparison between electric field intensity distribution inside the two multilayer stacks would show clearly the influence of an emitter’s polarization on the Purcell effect of multilayer stacks. Figure 5 shows the three-dimensional full-wave finite element simulation (COMSOL Multiphysics) result of the electric field intensity distribution inside both multilayer stacks from a dipole emitter near the multilayer top surface with an emission wavelength of 576 nm. The top and the bottom row shows the electric field intensity distribution of the dipole emitter with polarization parallel (*y*-polarized) and perpendicular (*z*-polarized) to the multilayer surfaces, respectively. The electric field intensity distribution of a *z*-polarized dipole inside multilayers is more spatially extended through the layers than that of a *y*-polarized dipole, which agrees with the fact that the major contribution to Purcell factor comes from the *z*-polarized dipole coupled to the multilayers^44^. For the *z*-polarized dipole emitter, its electric field intensity distribution inside the PM has a typical cone feature, whereas the one in the ThM multilayer is more extended through the metal-dielectric interfaces indicating a stronger interaction between the dipole emitter and the coupled SPPs modes.

## Conclusion

We have studied the Purcell effect of CdSe/ZnS QDs coupled to Au-SiO_2_ PM and ThM multilayer metamaterials, which possessing the same metal filling ratio but different lattice order. Larger Purcell factors have been demonstrated for the ThM multilayer in a broadband of emission wavelength of 70 nm by time-resolved photoluminescence decay measurements. Theoretical analysis also reveals that ThM provides stronger LDOS than PM does, covering the wavelengths ranging from elliptical to hyperbolic dispersion region. A stronger dipole emission electric field distribution inside ThM is shown at the wavelength of 576 nm, indicating a stronger coupling between the dipole and the coupled SPP modes. We also noticed through theoretical calculations that in ThM multilayers the nearest two layers to the emitter influence the Purcell factor mostly, however the entire ThM multilayers do contribute to the spontaneous emission enhancement process due to the cooperative mechanism between quasi-localization and coupled SPP mode interaction with the dipole emitter. The differences in dipole-multilayer coupling are purely due to the order of the layer arrangement inside the multilayer stacks. This work presents an extensive examination of the LDOS enhancement by an aperiodic multilayer metamaterial, which could extend the range of research activities on quasiperiodic metamaterial nanostructures with the potential of tailoring light-matter interactions at the nanoscale.

## Methods

### Sample preparation

An electron-beam evaporation system was used to deposit the multilayer stack on at the rate of 0.2 Å/sec for both Au and SiO_2_ layers. A variable angle spectroscopic ellipsometry (VASE, J. A. Woollam Co. VB400/HS-190) was used to characterize the optical constant of each material. We diluted the original CdSe/ZnS QDs solution (QDs weight ratio 3%, solvent: Chloroform) with the mixed solution of polymethyl methacrylate (PMMA weight ratio 2%, solvent: anisole), anisole, and chloroform, to a final volume ratio as PMMA: anisole: chloroform = 1:3:4, and a final weight ratio for QDs and PMMA as 0.05% and 0.2%, respectively. For the spin-coating procedure, the duration and spin speed were set as one minute and 1200 rotation-per-minute, respectively. Photoluminescence spectrum from the spin-coated QD-PMMA layer on a glass substrate is centered at 604 nm with full width at half maximum (FWHM) of 39 nm.

### Experimental characterization

Time-resolved photoluminescence measurements were conducted by using a time-correlated single photon counting system. This system consists of a picosecond-pulsed excitation source (emission wavelength 402 nm, pulse width of 52 ps) and a spectrometer (Horiba IHR550) equipped with a single photon counting module synchronized with the pulsed excitation.

## References

[1] Purcell E (1946). Spontaneous emission probabilities at radio frequencies. Physical Review.

[2] Purcell EM, Torrey H, Pound RV (1946). Resonance absorption by nuclear magnetic moments in a solid. Physical review.

[3] Haroche S, Kleppner D (1989). Cavity quantum electrodynamics. Physics Today.

[4] Walther H, Varcoe BT, Englert B-G, Becker T (2006). Cavity quantum electrodynamics. Reports on Progress in Physics.

[5] Wylie J, Sipe J (1985). Quantum electrodynamics near an interface. II. Phys. Rev. A.

[6] Buckley S, Rivoire K, Vučković J (2012). Engineered quantum dot single-photon sources. Reports on Progress in Physics.

[7] Birowosuto, M. D. *et al*. Fast Purcell-enhanced single photon source in 1,550-nm telecom band from a resonant quantum dot-cavity coupling. *Scientific reports* **2** (2012). 10.1038/srep00321 PMC3307054 22432053

[8] Lounis B, Orrit M (2005). Single-photon sources. Reports on Progress in Physics.

[9] Michler P (2000). A quantum dot single-photon turnstile device. Science.

[10] Tsakmakidis KL, Boyd RW, Yablonovitch E, Zhang X (2016). Large spontaneous-emission enhancements in metallic nanostructures: towards LEDs faster than lasers. Optics Express.

[11] Lozano G, Rodriguez SR, Verschuuren MA, Rivas JG (2016). Metallic nanostructures for efficient LED lighting. Light: Science & Applications.

[12] Le-Van, Q., Le Roux, X., Aassime, A. & Degiron, A. Electrically driven optical metamaterials. *Nat. Commun*. **7** (2016). 10.1038/ncomms12017 PMC4917961 27328976

[13] Shalaginov MY (2015). Enhancement of single-photon emission from nitrogen-vacancy centers with TiN/(Al, Sc) N hyperbolic metamaterial. Laser Photonics Rev..

[14] Ellis B (2011). Ultralow-threshold electrically pumped quantum-dot photonic-crystal nanocavity laser. Nature Photonics.

[15] Baba T, Sano D (2003). Low-threshold lasing and Purcell effect in microdisk lasers at room temperature. IEEE Journal of selected topics in quantum electronics.

[16] Wei, W., Yan, X. & Zhang, X. Ultrahigh Purcell factor in low-threshold nanolaser based on asymmetric hybrid plasmonic cavity. *Scientific Reports* **6** (2016). 10.1038/srep33063 PMC5018824 27616768

[17] Sorger VJ, Zhang X (2011). Spotlight on plasmon lasers. Science.

[18] Park, D. J. *et al*. Directional emission from dye-functionalized plasmonic DNA superlattice microcavities. *Proceedings of the National Academy of Sciences*, 201619802 (2017). 10.1073/pnas.1619802114 PMC5255591 28053232

[19] Droulias S, Jain A, Koschny T, Soukoulis CM (2017). Novel Lasers Based on Resonant Dark States. Physical Review Letters.

[20] Oulton RF (2009). Plasmon lasers at deep subwavelength scale. Nature.

[21] Reithmaier Já (2004). Strong coupling in a single quantum dot–semiconductor microcavity system. Nature.

[22] Vahala KJ (2003). Optical microcavities. Nature.

[23] Gérard J (1998). Enhanced spontaneous emission by quantum boxes in a monolithic optical microcavity. Physical review letters.

[24] Gérard J-M, Gayral B (1999). Strong Purcell effect for InAs quantum boxes in three-dimensional solid-state microcavities. Journal of lightwave technology.

[25] Nozaki K, Kita S, Baba T (2007). Room temperature continuous wave operation and controlled spontaneous emission in ultrasmall photonic crystal nanolaser. Optics express.

[26] Iwase H, Englund D, Vučković J (2010). Analysis of the Purcell effect in photonic and plasmonic crystals with losses. Optics express.

[27] Boroditsky M (1999). Spontaneous emission extraction and Purcell enhancement from thin-film 2-D photonic crystals. Journal of Lightwave technology.

[28] Noda S, Fujita M, Asano T (2007). Spontaneous-emission control by photonic crystals and nanocavities. Nature photonics.

[29] Belacel C (2013). Controlling spontaneous emission with plasmonic optical patch antennas. Nano Lett..

[30] Zhukovsky S (2014). Hyperbolic metamaterials based on quantum-dot plasmon-resonator nanocomposites. Opt. Express.

[31] Ozel T (2011). Anisotropic emission from multilayered plasmon resonator nanocomposites of isotropic semiconductor quantum dots. ACS Nano.

[32] Eggleston MS, Messer K, Zhang L, Yablonovitch E, Wu MC (2015). Optical antenna enhanced spontaneous emission. Proceedings of the National Academy of Sciences.

[33] Shelby RA, Smith DR, Schultz S (2001). Experimental verification of a negative index of refraction. Science.

[34] Wood B, Pendry J, Tsai D (2006). Directed subwavelength imaging using a layered metal-dielectric system. Phys. Rev. B.

[35] Orlov AA, Voroshilov PM, Belov PA, Kivshar YS (2011). Engineered optical nonlocality in nanostructured metamaterials. Phys. Rev. B.

[36] Iorsh I, Poddubny A, Orlov A, Belov P, Kivshar YS (2012). Spontaneous emission enhancement in metal–dielectric metamaterials. Phys. Lett. A.

[37] Subramania G, Fischer A, Luk T (2012). Optical properties of metal-dielectric based epsilon near zero metamaterials. Appl. Phys. Lett..

[38] Gao J (2013). Experimental realization of epsilon-near-zero metamaterial slabs with metal-dielectric multilayers. Appl. Phys. Lett..

[39] Maas R, Parsons J, Engheta N, Polman A (2013). Experimental realization of an epsilon-near-zero metamaterial at visible wavelengths. Nat. Photonics.

[40] Poddubny A, Iorsh I, Belov P, Kivshar Y (2013). Hyperbolic metamaterials. Nat. Photonics.

[41] Yang X (2013). Experimental demonstration of near-infrared epsilon-near-zero multilayer metamaterial slabs. Opt. Express.

[42] Sun L (2014). Experimental characterization of optical nonlocality in metal-dielectric multilayer metamaterials. Opt. Express.

[43] Sun L, Li Z, Luk TS, Yang X, Gao J (2015). Nonlocal effective medium analysis in symmetric metal-dielectric multilayer metamaterials. Phys. Rev. B.

[44] Li L, Wang W, Luk TS, Yang X, Gao J (2017). Enhanced Quantum Dot Spontaneous Emission with Multilayer Metamaterial Nanostructures. ACS Photonics.

[45] Shalaginov MY (2013). Broadband enhancement of spontaneous emission from nitrogen-vacancy centers in nanodiamonds by hyperbolic metamaterials. Appl. Phys. Lett..

[46] Jacob Z, Smolyaninov II, Narimanov EE (2012). Broadband Purcell effect: Radiative decay engineering with metamaterials. Appl. Phys. Lett..

[47] Ferrari L, Lu D, Lepage D, Liu Z (2014). Enhanced spontaneous emission inside hyperbolic metamaterials. Opt. Express.

[48] Galfsky T (2015). Active hyperbolic metamaterials: enhanced spontaneous emission and light extraction. Optica.

[49] Kim J (2012). Improving the radiative decay rate for dye molecules with hyperbolic metamaterials. Opt. Express.

[50] Krishnamoorthy HN, Jacob Z, Narimanov E, Kretzschmar I, Menon VM (2012). Topological transitions in metamaterials. Science (Washington, DC, U.S.).

[51] Lu D, Kan JJ, Fullerton EE, Liu Z (2014). Enhancing spontaneous emission rates of molecules using nanopatterned multilayer hyperbolic metamaterials. Nat. Nanotechnol..

[52] Moritake Y (2014). Lifetime reduction of a quantum emitter with quasiperiodic metamaterials. Physical Review B.

[53] Cheng F (2015). Enhanced structural color generation in aluminum metamaterials coated with a thin polymer layer. Optics Express.

[54] Malitson I (1965). Interspecimen comparison of the refractive index of fused silica. Josa.

[55] Johnson PB, Christy R-W (1972). Optical constants of the noble metals. Physical review B.

[56] Leistikow M, Johansen J, Kettelarij A, Lodahl P, Vos W (2009). Size-dependent oscillator strength and quantum efficiency of CdSe quantum dots controlled via the local density of states. Phys. Rev. B.

